# Preparation and characterization of ^10^B boric acid with high purity for nuclear industry

**DOI:** 10.1186/s40064-016-2310-6

**Published:** 2016-07-28

**Authors:** Weijiang Zhang, Tianyu Liu, Jiao Xu

**Affiliations:** School of Chemical Engineering and Technology, Tianjin University, No. 72, Weijin Road, Tianjin, 300072 China

**Keywords:** ^10^Boron trifluoride–methanol-complex, ^10^B boric acid, Electrical conductivity, ICP, Recrystallization

## Abstract

Boric acid is often added into coolant as neutron capture agent for pressurized water reactor, whose amount is influenced by its abundance and purity. Therefore, the preparation of enriched ^10^B boric acid with high purity is beneficial to nuclear industry. ^10^B is also used in developing tumor-specific boronated drugs in boron neutron capture therapy. The boronated drug can be administered to patient intravenously, intratumorally, or deposited at tumor site in surgical excision. Thus, enriched ^10^B boric acid is of practical significance in the field of medicine. Self-made boron trifluoride–methanol-complex solution was selected as one of the experimental reagents, and the preparation of ^10^B acid was realized by one-step reaction for the complexes with water and calcium chloride. The determination of electrical conductivity in reaction process proves that the optimum reaction time was 16–20 h. Furthermore, the effect of reaction time, ratio of calcium chloride to complex as well as the amount of water on the purity and yield of boric acid was investigated. Finally, the optimum reaction time was 20 h, the optimal solid–liquid ratio (molar ratio) was 3:1, and the amount of water was 1 L of deionized water for each mol of the complex. H_2_O_2_ was added in the reaction process to remove Fe^2+^. After recrystallization, IR spectra of ^10^B boric acid was measured and compared with standard to verify the product of boric acid. The feasibility of the preparation method was determined by the detection of XRD of boric acid. To observe the morphology by polarizing microscope, crystal structure was obtained. The purity of the final product is 99.95 %, and the yield is 96.47 %. The ion concentration of boric acid accords with the national standard of high purity, which was determined by ICP.

## Background

Boric acid (“Boric acid that appears in the following text refers to ^10^B boric acid.”) is widely used in nuclear power plant as coolant (moderator) for reactor to control nuclear reaction rate (Zhang et al. 2014, 2015). The important role of boric acid in nuclear power plant is to control nuclear fission rate and to influence the power generation of nuclear power plant (Odar 2007). Generally speaking, the rate of nuclear fission is controlled by neutron flux, which is released by that process, and boron is an atom that absorbs neutrons with perfect effect. ^10^B (one of B isotope) has very strong absorption ability, whose neutron absorption cross section of thermal neutron is five times more than boron with natural abundance, 20 times than graphite, and 500 times than traditional protective material of concrete. For that reason, the rate of nuclear fission can be controlled by injecting boric acid into a nuclear power furnace with ^10^B. Boric acid is prepared in a system consisting of boron and water supply, and enters into chemical volume control system by boron adding loop. The chemical volume control system controls the reactivity of the reactor by controlling boric acid concentration of the loop, and then further controls the power and safety of the reactor (Wootten et al. 1985; Mark 1989; Song and Lee 2003; Staudt et al. 2002).

The main advantages of boric acid solution for controlling water pressure reactor are (Pastina et al. 1999; Hohne et al. 2008): (1) boric acid features the property of dissolving in water. Since this property make boric acid’s effect on the reaction is uniform, and neutron absorption effect is well exerted without additional configuration space, a lot of control rods can be saved. Therefore, core arrangement and pressure vessel structure of the reactor on the top of the heap can be simplified. (2) Boric acid is chemically stable, and cannot react with the material of the loop easily, and its inertia is enhanced with the increase of temperature. Boric acid is neither being deposited itself, nor will be formed on the surface of the compound formed by the chemical composition of the reactor. (3) Boric acid can inhibit corrosion of reactor materials. Significant advantages have been shown by comparing enriched ^10^B acid with boric acid with natural abundance, since the increase of ^10^B concentration in reactor’s coolant system and the decrease of whole amount of boric acid can increase the safety and controllability of the reactor and improved, water chemical environment of coolant, and so on. For all the above reasons, the cost is effectively reduced.

Traditional preparation method of boric acid (Zheng 2000; Zhu et al. 2005; Li and Li 2008) is mostly to get the product with natural abundance and not high purity, whose impurities cannot meet the standard of high purity (Wang et al. 2014; Xue et al. 2015; Duan et al. 2012; Li et al. 2011). ^10^BF_3_–methanol complex, prepared by separation device of laboratory boron isotope, was chosen as raw material for preparing boric acid, and calcium chloride and distilled water play the same role. In the reaction process, Fe^2+^ was oxidized to form precipitation due to the addition of H_2_O_2_, and the product of boric acid was purified. Finally, the content of impurity ion was detected by ICP to confirm that final product of boric acid meets the requirement of the national standard of high purity.

## Experimental section

### Experimentation

1. Calcium chloride and deionized water were added into four flasks, being heated and stirred, and a certain amount of complex was weighted in a funnel and placed on the four flasks. The experimental device is shown in Fig. 1. When heating temperature was up to 85 °C, the complex was dropped, and the drop speed was controlled at rate of 0.5–1 drops/s.

**Fig. 1:**
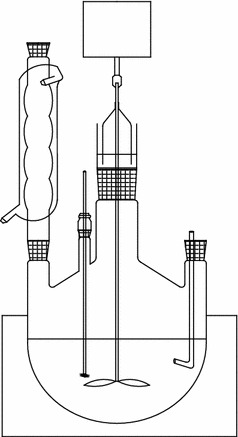
The schematic diagram of apparatus
2. After the completion of the complex, a certain amount of H_2_O_2_ was added into the same funnel to drop into the same four flasks with the same drop speed.
3. After the reaction, the reaction liquid was washed and filtered, and the filtrate was cooled and crystallized. The experimental apparatus of cooling crystallization for boric acid is shown in Fig. 2.

**Fig. 2:**
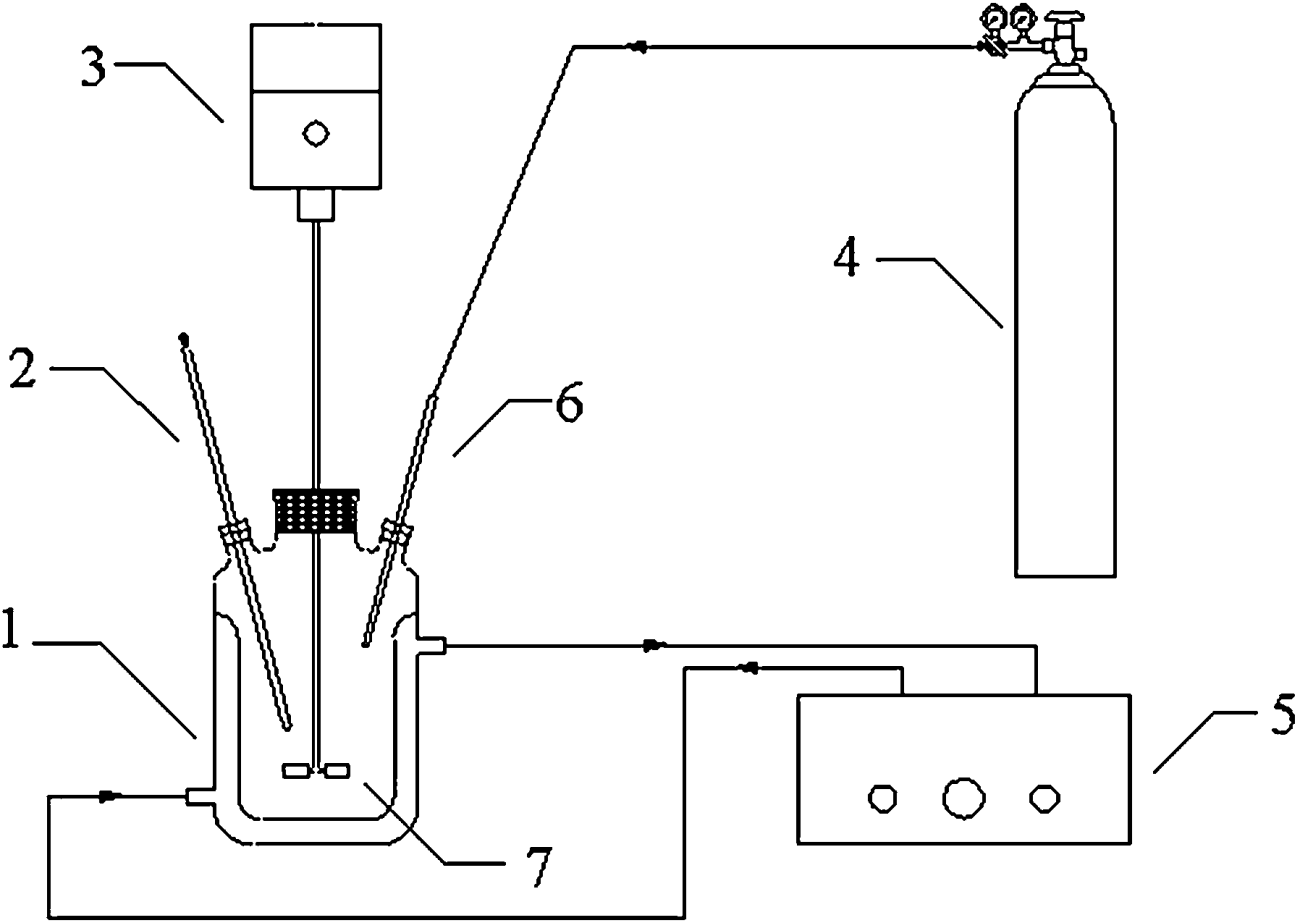
Schematic diagram of experimental apparatus for the cooling crystallization of boric acid: *1* crystallizer, *2* thermometer, *3* agitator motor, *4* nitrogen cylinder, *5* thermostat, *6* nitrogen tunnel, *7* impeller
4. Crystallization products were purified by recrystallization, and then their infrared spectra were determined. At the same time, their crystal morphology was observed by polarized light microscope.
5. The purity and yield of the final product boric acid were measured and calculated. The content of the impurity ion was detected by ICP, and then the purity of the product was compared with that of the national standard and with the boric acid with high purity produced in foreign countries.

### Detection method

“GB/T 628-2011” is the testing standard for boric acid’s purity in the experiment. Infrared spectrum and XRD were used in analyzing the product of boric acid. Ion concentration of the final product was detected by inductively coupled plasma (ICP).

## Results and discussion

In the experiment, the preparing process of boric acid by ^10^BF_3_–methanol complex, calcium chloride and deionized water as raw materials was studied. The complex and the mixed liquid of a certain amount of water and calcium chloride underwent heating reaction, and then were filtered through filter, dehydrated and dried, then boric acid solid was acquired. The effect of reaction time, raw material ratio and the amount of water on the yield and purity of boric acid was investigated. Optimum technological conditions were obtained.

### Determination of optimum reaction time and its effect on the purity and yield of boric acid

**Fig. 3:**
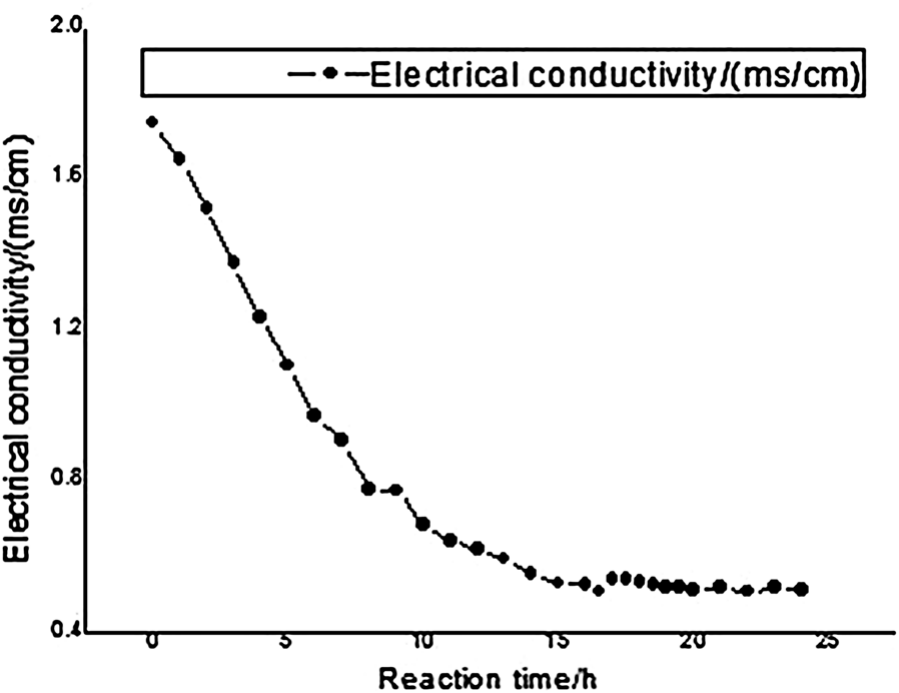
Effect of reaction time on the conductance

Electrical conductivity indicates the ability of solution to conduct electric current expressed in figures. Electrical conductivity of a kind of solution depends on the concentration of the solute or other chemical impurities that can decompose electrolyte. The conductivity of a kind of solution is an important index to measure the salt content of the solution, the composition of ions, the impurities and so on. The main components of the experimental solution include HBF_4_, HF, HBF_3_OH, HBF_2_(OH)_2_, HBF(OH)_3_ and H_3_BO_3_, and the acidity of the solution decreases according to HBF_4_ → HF → HBF_3_OH → HBF_2_(OH)_2_ → HBF(OH)_3_ → H_3_BO_3_. H_3_BO_3_ is a weak acid, essentially non ionizing, so the end of the reaction can be determined by electrical conductivity. The change of electrical conductivity in the reaction process is shown in Fig. 3.

By the graph, the reaction conductivity decreased with the increase of reaction time. At the beginning of the reaction, HF, HBF_3_OH, HBF_2_(OH)_2_ and HBF(OH)_3_ were generated, and the electrical conductivity was higher. As the reaction went on, boric acid was gradually formed, meanwhile HF, HBF_3_OH, HBF_2_(OH)_2_ and HBF(OH)_3_ gradually reacted completely. The electrical conductivity of boric acid is relatively low because it is a weak acid. With the increase of reaction time, the electrical conductivity of the system decreased. The change of the system’s electrical conductivity is basically flat after the reaction time was longer than 16 h, which has no change when it lasted for more than 20 h. Therefore, optimal reaction time was about 20 h.

The effect of reaction time on the purity and yield of boric acid was investigated in 16–24 h.

**Fig. 4:**
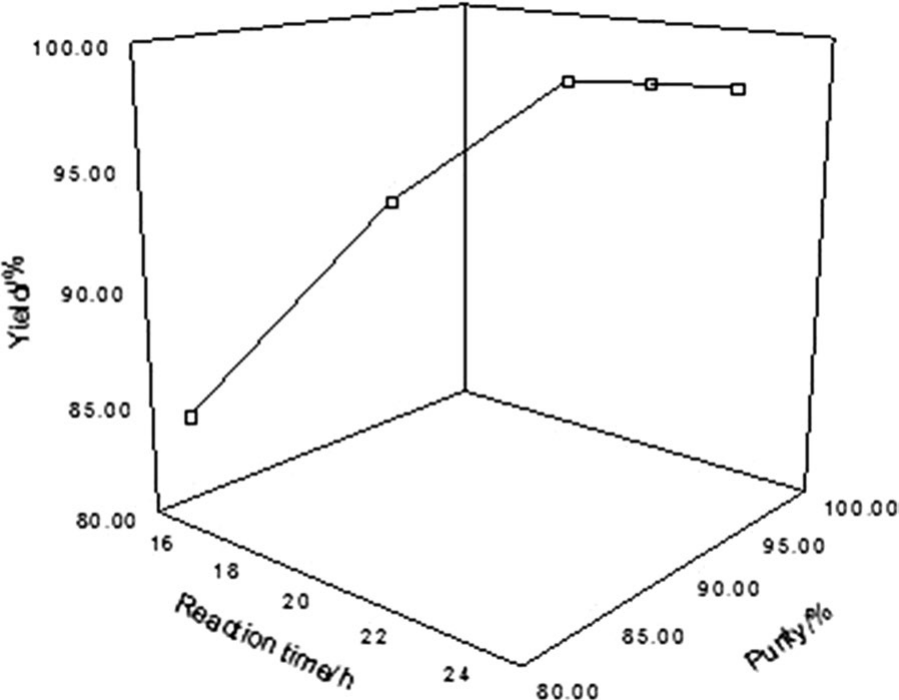
Effect of reaction time on the yield and purity of boric acid

From Fig. 4 we can see that with the increase of reaction time, the yield and purity of boric acid gradually increased. When reaction time was longer than 20 h, the yield and purity of boric acid reached the maximum, which would not increase any more even though the reaction was continued to carry on for a long time. Combined with Fig. 3, it clearly shows that electrical conductivity has no change after the reaction time was longer than 20 h. All the above prove that optimum reaction time is 20 h.

### Effect of solid–liquid ratio of reaction on the purity and yield of boric acid

**Fig. 5:**
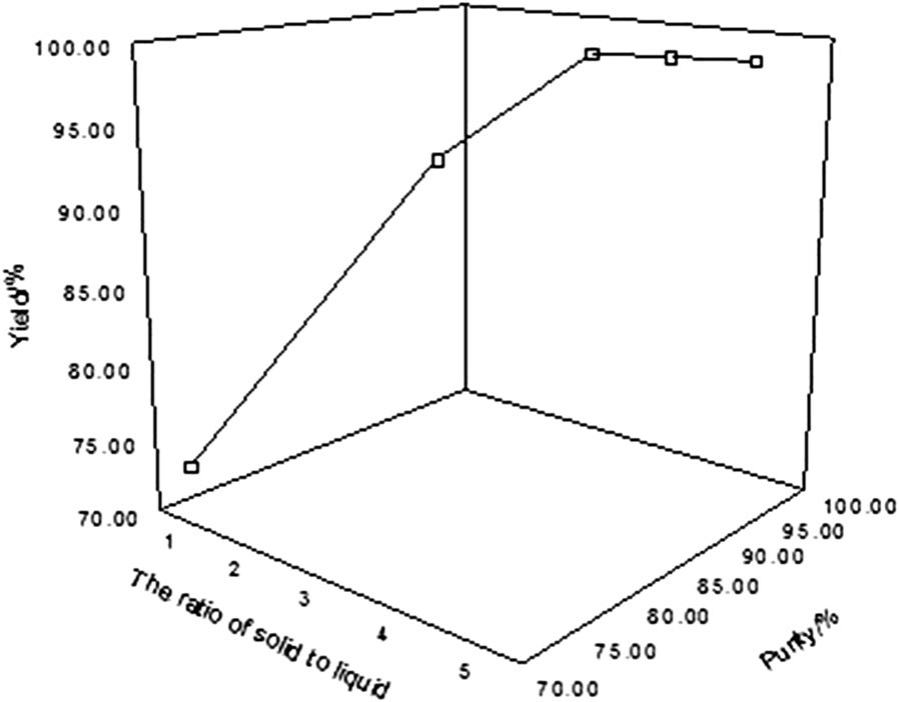
Effect of molar ratio of CaCl_2_ to BF_3_ on the yield and purity of boric acid

The effect of the ratio of raw materials between calcium chloride and ^10^BF_3_–methanol complex on the yield and purity of boric acid was investigated at the reaction temperature of 85 °C, reaction time being 20 h, and the amount of water was 500 g. The result is shown in Fig. 5.

The yield and purity of boric acid increased with the increase of the ratio of n(CaCl_2_):n(^10^BF_3_–methanol complex). When the ratio reached 3:1, the yield and purity of boric acid tended to balance, which basically did not increase even though the proportion of raw materials continued to increase. Accordingly, the ratio of n(CaCl_2_):n(10BF_3_–methanol complex) = 3:1 was appropriate.

### Effect of water amount on the purity and yield of boric acid

Water plays three important roles in the reaction process. Firstly, water is a kind of reactant, whose amount will affect whether the reaction is complete. Secondly, water, as dispersion medium, can make the formation of suspension with calcium chloride be dispersed in it. If water amount is in shortage, calcium chloride cannot be dispersed evenly. Lastly, water is a solvent for the production of boric acid. Once water amount is not enough, the product of boric acid will precipitate and mix with nonreacted calcium chloride, which might cause that it is hard to acquire pure boric acid. Water’s three roles testify that water amount should not be too less. However, there should not be too much water too. Otherwise, boric acid will be volatile with water in the concentration process of boric acid, reducing the yield of boric acid. At the same time, it will produce more liquid waste, which makes it more difficult to follow up.

**Fig. 6:**
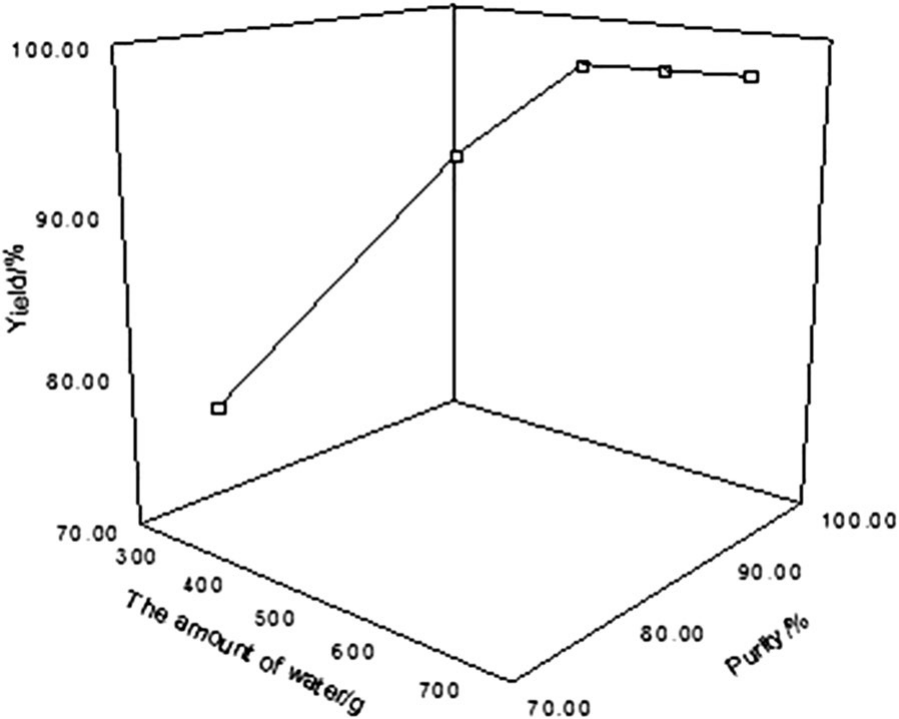
Effect of amount of water on the yield and purity of boric acid

The effect of water amount on the purity and yield of boric acid was investigated in the experiment under the condition of reaction temperature being 85 °C, reaction time being 20 h, calcium chloride being 150 g, and the ratio of calcium chloride and complex being 3:1. The result is shown in Fig. 6.

As can be seen from Fig. 6, the yield and purity of boric acid increases with the increase of water amount, and they keep unchanged even though it is more than 500 g. Under the circumstance, calcium chloride can be dispersed evenly and boric acid can be completely dissolved. Since theoretical molar ratio of calcium chloride and water is 1:1.5, this water amount is fully able to meet the needs of the reaction. Therefore, 500 g of water amount is appropriate for the experiment.

### Effect of added amount of H_2_O_2_ on the concentration of Fe^2+^ in boric acid

In boric acid solution, Fe^2+^ can be oxidized, and the reaction equation is as follows:

$$2{\text{Fe}}^{2 + } + {\text{H}}_{2} {\text{O}}_{2} + 2{\text{H}}^{ + } \to 2{\text{Fe}}^{3 + } \downarrow + 2{\text{H}}_{2} {\text{O}}$$

K_sp_(Fe(OH)_2_) = 4.86 × 10^−17^, K_sp_(Fe(OH)_3_) = 2.8 × 10^−39^. Fe^2+^ will be converted to Fe^3+^ by oxidation and Fe^3+^ in the solution was completely precipitated. When c(Fe^3+^) ≤10^−5^ mol/L, pH ≥3.2 (Wang et al. 2009), oxidation products precipitated by changing pH, which was 4–5 in boric acid solution, and impurities were removed by thermal filtration. The amount of added H_2_O_2_ was 3 % of the solution volume, which can effectively reduce the concentration of Fe^2+^ in the product of boric acid. Specific numerical analysis was carried out for the impurity ion in boric acid.

### IR spectra analysis of boric acid

**Fig. 7:**
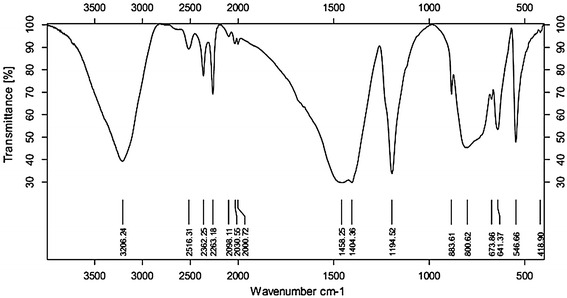
Infrared spectra of boric acid products

Boric acid acquired from the experiment was analyzed for IR spectra, using KBr compression method, and the results of infrared spectrum are shown in Fig. 7.

The stretching vibration of intermolecular hydrogen bond (O–H) is between 3500 and 3200 cm^−1^, characteristic peaks of B–O bond is between 1430 and 1355 cm^−1^, the stretching vibration of C–O is between 1300 and 1000 cm^−1^, and surface curvature of O–H is between 769 and 659 cm^−1^. Upon comprehensive analysis and comparison with standard spectra, the product obtained was boric acid.

### Analysis of X-ray powder diffraction

**Fig. 8:**
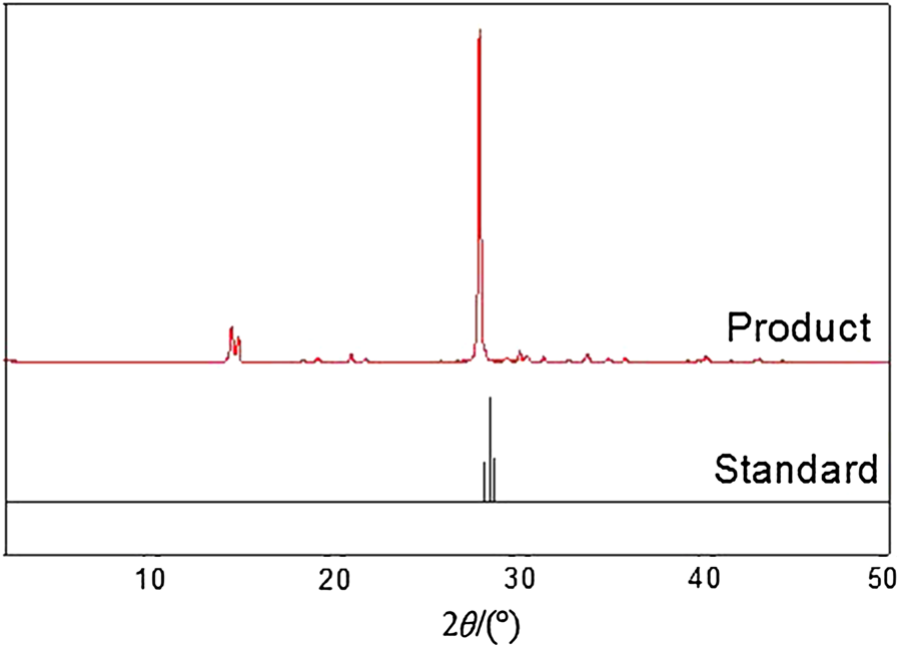
The X-ray diffraction pattern and the X-ray diffraction standard pattern for the boric acid

The crystal obtained by crystallization purification (with purity being 99.95 %) was analyzed by X-ray powder diffraction. The pattern of the determination and the standard were basically the same, which confirms with that borate crystal can be obtained by that method. What can be seen from the figure is that boric acid products are impurities. The specific quantitative analysis of the impurities needs to be performed by ICP, which will be discussed in the next section. X-ray powder diffraction pattern of boric acid is shown in Fig. 8.

### Crystal structure diagram of boric acid

**Fig. 9:**
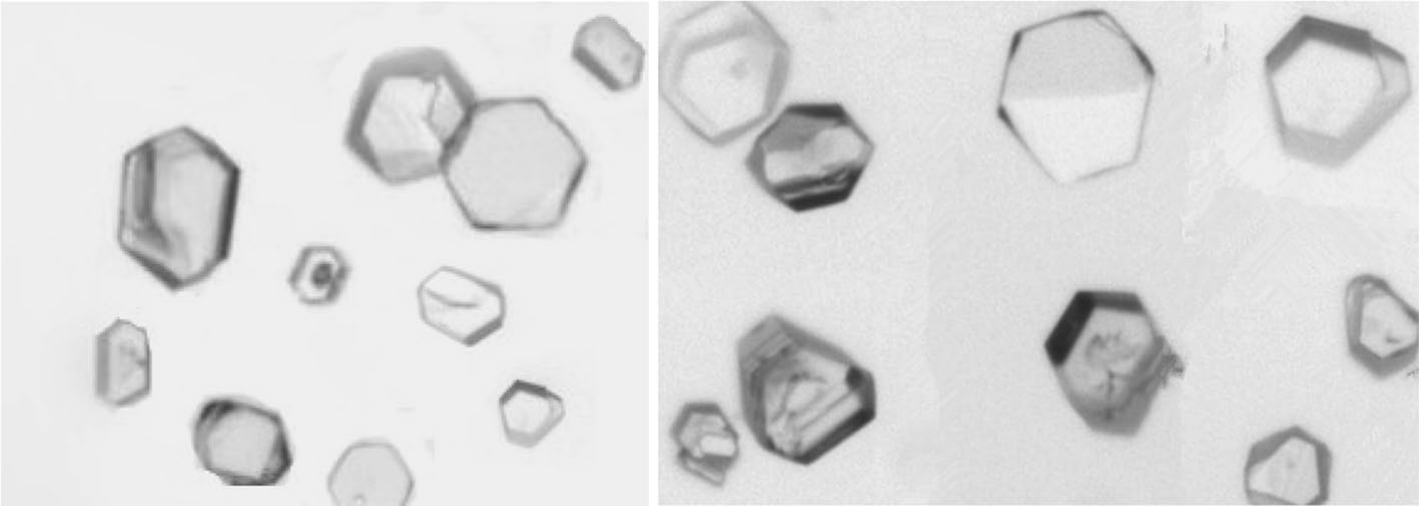
Microscope images of enriched ^10^B boric acid product

Polarizing optical microscope is a kind of optical microscope, which can be used to study the morphology and structure of transparent and opaque materials. Polarizing optical microscope puts polarizing film on ordinary optical microscope, which only allows the light vibrated at certain direction passing through. Polarized light microscope is a tool for studying the structure of crystal, and can be used to characterize crystal structure. The crystal structure of boric acid by one-step preparation was observed by using polarized light microscope in the experiment, and the structure of boric acid crystal is shown in Fig. 9.

### Detection of the concentration of the impurity ion in boric acid

The product of boric acid was purified by recrystallization. The purity of the product can reach 99.95 % and the yield reaches 96.47 % with purification twice. The ion content of boric acid was detected by ICP. Compared with analytical reagent of boric acid and boric acid with high purity in “GB/T 628-2011”, the same with major ion concentration of boric acid with high purity produced by foreign company (E. Merck) (Ministry of Chemical Industry 1995). The results are shown in Table 1.

**Table 1:** Ion concentration detection of optimum product and the results of comparison

Ion	Sample in this study/10^−6^	Analytical purity sample/10^−6^	High purity sample of GB/T 628-2011/10^−6^	High purity sample of E. Merck/10^−6^
(Cl)	2.1	5	5	1
(Ca)	0.6	20	2	0.05
(Fe)	1.2	5	0.5	0.05
(Fe)	5.4 (without adding H_2_O_2_)			
(Pb)	<0.005	10	0.2	0.02
(As)	0.03	1	0.5	0.05
(SO_4_)	0.5	20	20	5
Undissolved substance	10	50	50	–

As can be seen from Table 1:

1. The standard of main ion concentration of boric acid is much higher than that of national analytical reagent, which meets the requirement of national high purity.
2. With adding H_2_O_2_, the concentration of Fe^2+^ in the solution is significantly reduced. It can be clearly seen from the data that the addition of H_2_O_2_ make the products meet the requirements of the national analytical reagent. The advantage of adding H_2_O_2_ is obviously.
3. Compared with the standard of boric acid with high purity produced by foreign companies, domestic production technology is still to be improved, which has a certain gap with the world’s leading boric acid production technology, requiring developing more advanced technology of boric acid purification.

## Conclusion

1. The optimum reaction condition for preparing boric acid with ^10^BF_3_–methanol complex, deionized water and calcium chloride as raw materials are as follows: optimum reaction time is 20 h, the ratio of n(calcium chloride) and n(10BF_3_–methanol complex) is 3:1, and water amount is 1 L deionized water per mol complex. Under this condition, the purity of ^10^B boric acid can reach 99.95 % and the yield reaches 96.47 %.
2. It is proved that the product is boric acid and the production is feasible by the analysis of IR and XRD. The crystal structure of boric acid is observed and the experimental results are real and reliable.
3. The addition amount of H_2_O_2_ is 3 % of the solution volume, which can effectively reduce the concentration of Fe^2+^ in the product of boric acid.
4. The concentration of the impurity ion in the product of boric acid meets the requirement of national high purity standard by detection of ICP after purification by recrystallization, which can be used in nuclear industry.
